# Cardiac Function and Architecture Are Maintained in a Model of Cardiorestricted Overexpression of the Prorenin-Renin Receptor

**DOI:** 10.1371/journal.pone.0089929

**Published:** 2014-02-25

**Authors:** Hasan Mahmud, Wellington Mardoqueu Candido, Linda van Genne, Inge Vreeswijk-Baudoin, Hongjuan Yu, Bart van de Sluis, Jan van Deursen, Wiek H. van Gilst, Herman H. W. Silljé, Rudolf A. de Boer

**Affiliations:** 1 University of Groningen, University Medical Center Groningen, Department of Cardiology, Groningen, The Netherlands; 2 University of Groningen, University Medical Center Groningen, Department of Molecular Genetics, Groningen, The Netherlands; 3 Department of Pediatric and Adolescent Medicine, Mayo Clinic, Rochester, Minnesota, United States of America; 4 Department of Biochemistry and Molecular Biology, Mayo Clinic, Rochester, Minnesota, United States of America; Max-Delbrück Center for Molecular Medicine (MDC), Germany

## Abstract

The (pro)renin-renin receptor, (P)RR has been claimed to be a novel element of the renin-angiotensin system (RAS). The function of (P)RR has been widely studied in renal and vascular pathology but the cardio-specific function of (P)RR has not been studied in detail. We therefore generated a transgenic mouse (Tg) with cardio-restricted (P)RR overexpression driven by the alpha-MHC promotor. The *mRNA* expression of *(P)RR* was ∼170-fold higher (P<0.001) and protein expression ∼5-fold higher (P<0.001) in hearts of Tg mice as compared to non-transgenic (wild type, Wt) littermates. This level of overexpression was not associated with spontaneous cardiac morphological or functional abnormalities in Tg mice. To assess whether (P)RR could play a role in cardiac hypertrophy, we infused ISO for 28 days, but this caused an equal degree of cardiac hypertrophy and fibrosis in Wt and Tg mice. In addition, ischemia-reperfusion injury was performed in Langendorff perfused isolated mouse hearts. We did not observe differences in parameters of cardiac function or damage between Wt and Tg mouse hearts under these conditions. Finally, we explored whether the hypoxia sensing response would be modulated by (P)RR using HeLa cells with and without (P)RR overexpression. We did not establish any effect of (P)RR on expression of genes associated with the hypoxic response. These results demonstrate that cardio-specific overexpression of (P)RR does not provoke phenotypical differences in the heart, and does not affect the hearts’ response to stress and injury. It is concluded that increased myocardial (P)RR expression is unlikely to have a major role in pathological cardiac remodeling.

## Introduction

The (pro)renin-renin receptor, (P)RR, was discovered and cloned in 2002 as a novel element of the renin-angiotensin system (RAS) [1]. Stimulation of (P)RR, via (pro)renin or indirectly, has been suggested to play a role in organ damage, for instance in blood vessels and the kidney. (P)RR is expressed in kidney, specifically in renal mesangial cells, brain, vascular smooth muscle cells and blood vessels, in macrophages, T cells, granulocytes, and also, albeit at low levels, in the heart [2]. It has been suggested that (P)RR confers signals from an activated (tissue) RAS [3]–[5]. But (P)RR also has specific other functions in the assembly and function of vacuolar H^+^-ATPase (V-ATPase), an ATP-dependent proton pump which acidifies intracellular compartments [6].

Binding of both renin and its inactive precursor, prorenin, to the (P)RR exerts effects via angiotensin II-independent pathways, e.g.via second messengers including mitogen-activated protein kinases (MAPK) [2]. This has been shown to be associated with increased cell proliferation and upregulation of profibrotic genes [7]. Animal studies have suggested that (P)RR overexpression causes nephropathy and hypertension as well as activation of RAS [8]–[10]. It has been suggested that increased expression of (P)RR may be involved in renal and cardiac pathophysiology [7], [11]–[14].

The pathophysiological role of (P)RR was mainly investigated based on analyses of an animal model of ubiquitously (P)RR overexpression [8], [9] and a rat model of (P)RR overexpression in vascular smooth muscle cells, which resulted in elevated blood pressure and aldosterone levels [15]. In the heart however, the phenotypic consequences of altered (P)RR signaling are less clear. Cardiomyocyte-specific deletion of (P)RR causes a fulminant cardiomyopathy, an effect largely attributable to the impaired acidification due to the V-ATPase function of (P)RR [6]. We have shown that (P)RR expression is increased in murine, rat and human heart failure suggesting that (P)RR may play a role in cardiac remodeling [11]. A very recent study has indicated that (P)RR overexpression by adenovirus mediated gene delivery resulted in enhanced matrix remodeling [16]. Therefore considerable interest exists to study the functional consequences of (P)RR expression and activation in the heart as the precise role of (P)RR, if any, in cardiac disease remains unclear.

In the present study, we investigated the role of (P)RR by generating a mouse with cardiorestricted (P)RR overexpression, and evaluated its functional consequences in cardiac remodeling and dysfunction. Furthermore, we also examined the role of (P)RR in ischemia-reperfusion injury. We report that cardio-specific overexpression of (P)RR did not provoke phenotypical differences, and did not affect the hearts’ response to stress and injury. Therefore, based on this study, it is suggested that (P)RR has no or marginal contribution to the cardiac remodeling process.

## Materials and Methods

### Animal Model and Experimental Protocols

#### Generation of a transgenic mouse with cardio-restricted (P)RR overexpression

The murine (P)RR gene (Gene bank: NM_027439) was amplified by polymerase chain reaction (PCR) using primers containing SalI and HindIII restriction sites (Table S1). The PCR product was cloned into a previously described vector containing the cardio-specific α-MHC promoter [17], [18]. The BamHI fragment of this construct, containing α-MHC promoter and downstream (P)RR cDNA sequence, was subsequently used for pronuclear injections to generate a transgenic mice with cardiorestricted (P)RR overexpression in a FVB background. After multiple pronuclear injections, we succeeded to generate one transgenic mouse line; a total of 22 mouse lines turned out to be non-transgenic. The transgenic mice were subsequently back-crossed with C57BL/6J mice. Transgenic offspring were identified by PCR amplification of the transgene. Male transgenic (Tg) mice were used for all the experiments and non-transgenic littermates were used as wild type (Wt) controls for comparison with Tg mice.

### Ethics

The protocols describing the animal experiments were approved by the Animal Ethical Committee of the University of Groningen, the Netherlands, and conforms with the Guide for the Care and Use of Laboratory Animals published by the Directive 2010/63/EU of the European Parliament.

### Perturbations

#### Isoproterenol induced cardiac remodeling

Male mice, aged 8–12 weeks (n = 7) were allocated to two groups, a saline group and isoproterenol (ISO) group. All solutions were continuously infused for 28 days via osmotic minipumps (Charles River), as described previously [19]. In order to induce cardiac hypertrophy, mice were treated with ISO (Sigma Aldrich, 35 mg/kg/d) in saline with 0.1% ascorbic acid. Saline-treated animals received saline and 0.1% ascorbic acid as control. Briefly, the minipumps were implanted in the left flank of the mice under isoflurane (2% in air) anesthesia. At the end of the treatment animals were sacrificed, hearts were weighed and collected for molecular analysis.

#### Langendorff isolated heart perfusion

Experiments were performed with male mice, aged 8–12 weeks (n = 6). Hearts were isolated and perfused in the Langendorff mode (60 mmHg, 37°C) using a commercially available Langendorff set-up (Harvard Apparatus), as describe previously [20]. Briefly, mice were anesthetized with isoflurane; heparin (200 IU/Kg) was injected to prevent coagulation. Hearts were excised and immediately placed in cold (4°C) Krebs Henseleit bicarbonate buffer solution (KH, in mM): 118 NaCl, 11 glucose, 4.7 KCl, 24 NaHCO_3_, 2 CaCl_2_, 0.1 pyruvate, 0.5 glutamine, 1 lactate and 1.2 MgSO_4_. The aorta was rapidly cannulated and the hearts were perfused in a retrograde mode. The KH buffer was saturated with 95% O_2_ and 5% CO_2_ at 37°C providing a pH of 7.4.

A water-filled balloon was inserted through the left atrium into the left ventricle and adjusted to achieve a left ventricular end-diastolic pressure (LVDEP) of ∼5–10 mmHg, and equilibrated for 15 minutes. Left ventricular developed pressure (LVDP) was measured with an intraventricular balloon catheter attached to a computerized bridge amplifier during the entire experiment. After 15 minutes of equilibration, global ischemia was induced by stopping the flow of KH-buffer over the heart for 45 minutes, and then the flow was restarted to create 30 minutes of reperfusion. Heart function was measured at various times points of the protocol.

#### Heart functional measurements

The left ventricular developed pressure (LVDP) and rate pressure product (RPP) were calculated as LVDP = left ventricular systolic pressure (LVSP) – left ventricular end diastolic pressure (LVEDP); RPP = (heart rate (HR)×LVDP).

### Echocardiography and Blood Pressure Measurements

Cardiac function was assessed by echocardiography at baseline and prior to sacrifice with Vivid 7 (GE Healthcare, Chalfont St Giles, UK) equipped with a 13-MHz (mice) phase array linear transducer), as described previously [21]. The echocardiographic measurements were performed under general anesthesia with 2% isoflurane. Both 2-dimensional (2D) images in parasternal long-axis and short-axis view and 2-D guided M-mode tracing were obtained.

Furthermore, prior to sacrifice, blood pressure was measured, using an indwelling pressure tip catheter (Millar Instruments, Houston, TX, USA), that is introduced in the right carotid artery and advanced into the LV as described previously [21]. Blood pressure was recorded in the aortic arch, proximal to the aortic constriction.

### Masson Trichrome and Hematoxylin Staining

Masson’s trichrome staining was performed on paraffin sections of hearts from all experimental animals. Whole stained sections were scanned by a scanning system (ScanScope, Aperio Technologies, Vista, CA, USA). Total fibrosis was calculated automatically by the software at a 20× magnification for the entire section and expressed as percentage of total area, as described previously [22]. Paraffin sections were stained with Hematoxylin and Eosin (H&E) for gross appearance of the hearts and whole stained sections were scanned by a scanning system (ScanScope, Aperio Technologies, Vista, CA, USA).

Furthermore, to quantify individual myocyte size, paraffin sections were stained with isothiocyanate-conjugated wheat germ agglutinin (FITC-WGA) as described previously [23]. Myocyte area was assessed 20× magnification and analyzed with Image J software. Average myocyte area was evaluated by measurement of ∼400 cells per group of mice.

### Western Blot Analysis

A total of 35 µg of whole tissue protein lysates were separated on a SDS-PAGE gel and proteins were immunoblotted onto nitrocellulose membranes. Membranes were incubated with goat polyclonal (P)RR primary antibody (Novus Biological, catalogue# NB100-1318, 1 µg/mL) or with phospho-ERK mouse monoclonal antibody (Santa Cruz, catalogue#SC7383, 1∶1000 dilution), or with ERK(1/2) rabbit polyclonal antibody (Cell Signaling, catalogue#4695, 1∶1000) or with phospho-p38 rabbit polyclonal antibody (Cell Signaling, catalogue#9211, 1∶1000) or with p38 rabbit polyclonal antibody (Cell Signaling, catalogue#9212, 1∶1000) or with anti-Hsp47 rabbit monoclonal antibody (Abcam, catalogue#ab109117, 1∶1000) or with rabbit monoclonal GAPDH primary antibody (400 ng/mL, Fitzgerald Inc.). After incubation with secondary polyclonal rabbit anti-goat (Dako Inc. 1∶4000) or horse anti-mouse (Cell signaling, catalogue#7076, 1∶1000) or polyclonal goat anti-rabbit (1∶2000) antibodies, proteins were detected using Super Signal West Pico Chemiluminescent Substrate (Thermo Scientific Inc.). The band densities were scanned and quantified using a Bio Imaging (Syngene, Cambridge, United Kingdom) device and normalized to GAPDH.

### Measurement of Lactate Dehydrogenase (LDH) and Creatine Kinase (CK)

Samples of coronary effluent were collected from isolated perfused mouse heart at the following time points: 1) the end of a 20 minutes stabilization period, 2) immediately after 45 minutes of global ischemia, 3) after 5 minutes reperfusion, 4) after 10 minutes reperfusion, and 5) after 30 minutes reperfusion. After collection, samples were frozen at −80°C. The LDH (Cayman Chemical Co.) and CK (Bio Assay Systems) assays were performed using a spectrophotometer, according to the manufacturers’ protocol.

### Cell Culture and Induction of Hypoxia

HeLa S3 cells were cultured in DMEM with 4.5 g/L glucose supplemented with 10% FCS, 100 U/mL penicillin and 100 µg/mL streptomycin. Cells were grown in a humidified incubator at 5% CO_2_ and 37°C. For plasmid transfection of HeLa S3 cells, lipofectamine LTX reagent (Invitrogen) was used. Cells in a 12-well plate at 60–80% confluency were transfected with 0.5 µg DNA for 24 h. For induction of hypoxia, cells were either kept in an air tight with a pouch (Gaspak, Becton Dickinson) without oxygen or incubated with hypoxia mimetic deferoxamine (100 µM, Sigma Aldrich) for 24 hours at 37°C.

### mRNA Expression and Quantitative RT-PCR

Relative gene expression in left ventricular tissue of *(P)RR,* atrial natriuretic peptide *(ANP),* brain natriuretic peptide (*BNP*), galectin-3 (*gal-3*), collagen-1 (*col-1*), collagen-3 (*col-3*), and monocyte chemotactic protein-1 (*MCIP1*), were determined by real time (RT)-PCR and expressed relative to *36B4*, as described previously [24]. In the cell experiments, adrenomedullin (*ADM*), vascular endothelial growth factor (*VEGF*), *c-jun and c-fos* gene expression levels were determined by real time (RT)-PCR and expressed relative to *GAPDH*. Values were expressed relative to appropriate control groups. The primers used for the RT-PCR are listed in Table S1.

### Statistical Analysis

Data are expressed as means ± standard errors of the mean (SEM). Differences between two groups were tested using Student’s *t*-test and between multiple groups using ANOVA, and results were considered statistically significant when *P*-values were <0.05.

## Results

### Baseline Characteristic of the (P)RR Overexpression Transgenic Mice

**Figure 1:**
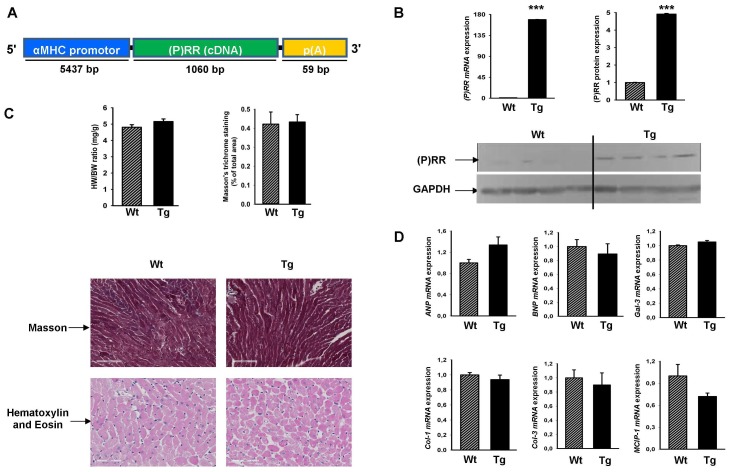
Generation of (P)RR transgenic mice and baseline characteristics. A) Schematic depiction of the generation of Tg mice with cardiomyocyte-restricted (P)RR overexpression. We cloned the 1060 bp murine (P)RR cDNA in front of the a 5.5-kb α-MHC promoter. B) (P)RR mRNA expression normalized to 36B4 by RT-PCR of Tg mice and their Wt littermates (left upper panel); western blot for (P)RR and GAPDH protein from hearts of Tg and Wt mice (bottom panel) and quantification of the western blot (right upper panel). C) Heart-weight (HW) normalized to body weight (BW) in adult Wt and Tg mice (left upper panel); images for Masson staining for quantification of fibrosis (bar size 100 µm) and haematoxylin and Eosin staining (bar size 70 µm) for assessment of cardiac morphology (bottom pannels) and quantification of Masson staining (right upper panel). D) mRNA expression of *ANP*, *BNP*, *Gal-3*, *Col-1*, *Col-3,* and *MCIP1* (normalized to *36B4*) of the Wt and Tg mice. Fold changes are shown. *** *P<0.001*, Wt vs. Tg.

The α-MHC-(P)RR construct is shown in figure 1A and was used to create the Tg mice. mRNA and protein (P)RR expression was studied in the whole heart. The *mRNA* expression of *(P)RR* was about 170 fold higher (figure 1B) and protein expression of (P)RR was 5 fold higher (figure 1B) in hearts of Tg mice, as compared to non-transgenic (wild type, Wt) littermates. To examine the potential role of (P)RR in the heart, we analyzed the heart weight (HW) of these animals at 8-weeks of age and did not observe differences between Wt and Tg mice, as determined by Masson Trichrome (fibrosis) and Hematoxylin-Eosin (cell appearance) staining (figure 1C). No obvious morphological abnormalities were observed in the hearts from Tg mice (figure 1C). To further study the effect of (P)RR overexpression on the heart, we analyzed a list of cardiac genes associated with cardiac hypertrophy and fibrosis, but did not observe differences between Wt and Tg mice (figure 1D).

### (P)RR Exerts no Effects in ISO-induced Cardiac Hypertrophy

**Figure 2:**
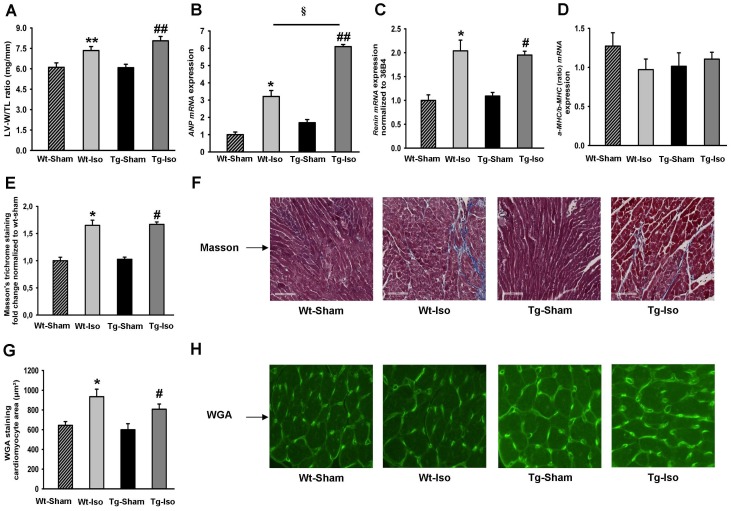
Assessment of isoproterenol induced cardiac hypertrophy in (P)RR transgenic mice. A) Left-ventricular weight (LV-W) to tibia length (TL)in adult Wt and Tg mice subjected to saline or ISO infusion for 28 days (N = 7 mice in each group). B) *ANP* mRNA expression (normalized to *36B4*) in adult Wt and Tg mice subjected to saline or ISO infusion for 28 days (N = 7 mice in each group). C) Renin mRNA expression (normalized to *36B4*) in adult Wt and Tg mice subjected to saline or ISO infusion for 28 days (N = 7 mice in each group). D) *α-MHC/β-MHC* (ratio) mRNA expression (normalized to *36B4*) in adult Wt and Tg mice subjected to saline or ISO infusion for 28 days (N = 7 mice in each group). E and F) Masson’s trichrome staining (bar size 100 µm) to assess myocardial fibrosis in adult Wt and Tg mice subjected to saline (sham procedure) or ISO infusion for 28 days; E: quantification, and F: typical examples of Masson’s trichrome staining. G and H) FITC-WGA staining to assess myocyte hypertrophy in adult Wt and Tg mice subjected to saline (sham procedure) or ISO infusion for 28 days; G: quantification, and H: typical examples of FITC-WGA staining. *P<0.05, **P<0.01, sham vs. ISO between Wt mice; ^#^ *P*<0.05, ^##^ *P*<0.01, sham vs. ISO between Tg mice and ^§^ *P*<0.05, ISO treatment between Wt and Tg mice.

Since we did not observe a spontaneous phenotype in the (P)RR Tg mice, we conducted experiments to stress the heart. To assess whether (P)RR plays a role in cardiac hypertrophy, we infused ISO in both Tg and Wt mice. ISO in a dose of 35 mg/kg/BW for 28 days resulted in marked cardiac hypertrophy, as determined by the increase in left-ventricular weight (LV-W) to tibia length (TL), but no difference was observed between the groups (figure 2A). The expression of the hypertrophic marker *ANP* was significantly increased by ISO in Tg and Wt mice (figure 2B). ISO infusion also significantly increased the expression of renal renin *mRNA* in both Tg and Wt groups (figure 2C) with no differences between groups. No differences were observed for *α-MHC/β-MHC* ratio between the groups (figure 2D). Changes in extracellular matrix (ECM) may affect cardiac function and therefore we performed Masson staining to quantify the ECM deposition. As shown in figure 2E and 2F, the ECM deposition was similarly increased in both Wt and Tg mice treated with ISO without differences between groups. Myocyte surface area determined by FITC-WGA staining, a measure of myocyte hypertrophy, was also increased by ISO in both Tg and Wt mice, but no difference was observed between the groups (figure 2G and 2H).

**Figure 3:**
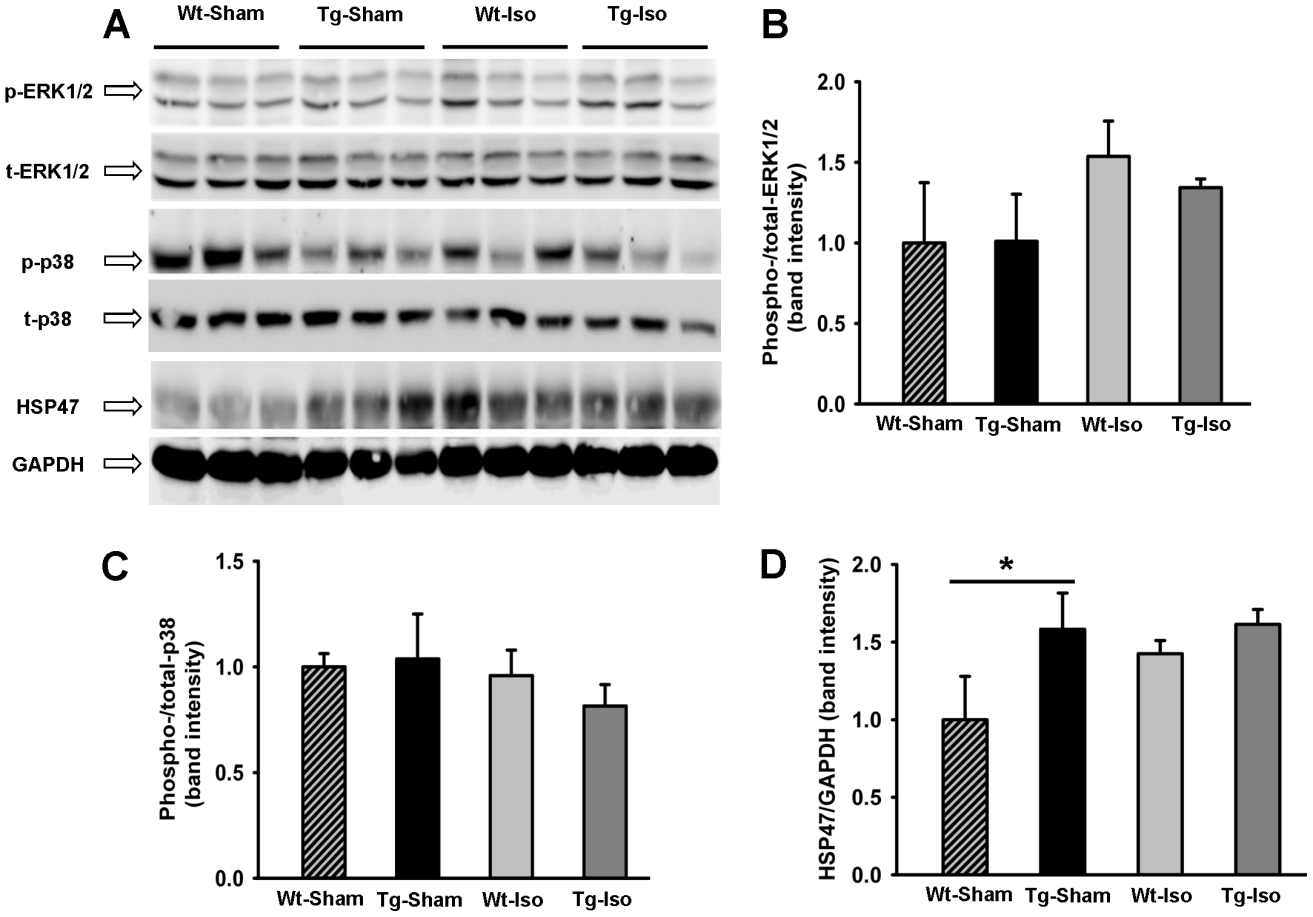
Western blot of ERK1/2, p-38 and HSP47. **A)** Western blot of the expression of ERK1/2, p-38 and HSP47 proteins from hearts of Tg and Wt mice with and without ISO treatment. B) Quantification of the ratio of phospho−/total Erk1/2 from hearts of Tg and Wt mice with and without ISO treatment. C) Quantification of the ratio of phospho−/total p-38 from hearts of Tg and Wt mice with and without ISO treatment. D) Quantification of the HSP47 protein expression from hearts of Tg and Wt mice with and without ISO treatment. * *P<0.05*, Wt vs. Tg.

To investigate the expression of stress related proteins which were previously linked to (P)RR signaling, we assessed protein expression of ERK1/2, p38 and HSP47 by western blot (figure 3A) in the heart of saline and ISO treated Wt and Tg mice. The ratio of phospho−/total Erk1/2 was not significantly different between groups (figure 3B) and the ratio of phospho−/total p38 was identical for each group (figure 3C). These observations are in line with the study with Taglieri DM *et al*. [25]. Furthermore, HSP47 protein expression was significantly higher in Tg mice at baseline and ISO stimulation did not affect this (figure 3D). These data suggest that myocardial overexpression of (P)RR does not influence the stress related proteins ERK1/2 and p38 with or without ISO treatment, and may influence HSP47 under control conditions, but not with ISO treatment.

Echocardiography was performed before sacrificing the animals (data shown in Table S2). Interventricular septum width (IVSd) and LV Posterior Wall thickness (LVPWd) were both increased in Wt and Tg mice upon ISO treatment, confirming cardiac hypertrophy. No substantial functional differences were observed. Furthermore, blood pressure measurements were performed at sacrificing the animals (data shown in Table S2). Mean arterial pressure (MAP) measured in the aortic arch was significantly higher in the Wt mice at baseline. Systolic blood pressure (SBP), diastolic blood pressure (DBP) and mean arterial pressure (MAP) were significantly increased in Tg mice upon ISO treatment. No substantial differences were observed between ISO treated Wt and Tg mice.

### Cardiomyocyte-restricted (P)RR Overexpression and Ischemia Reperfusion Injury

**Figure 4:**
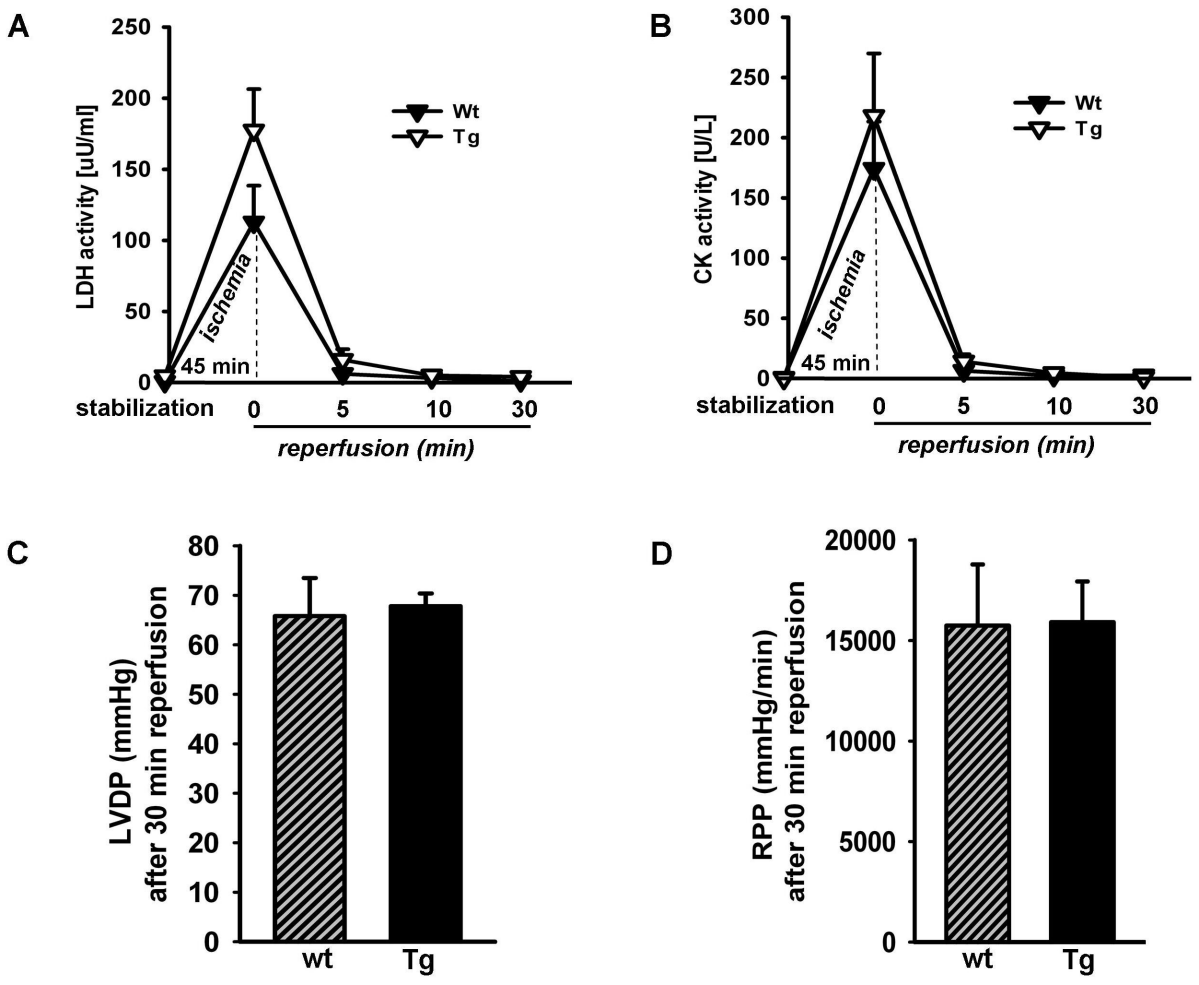
Assessment of ex vivo ischemia reperfusion injury by Langendorff isolated heart perfusion in (P)RR Tg mice. A) Leakage of lactate dehydrogenase (LDH) before and after ischemia and 5, 10 and 30 min after reperfusion of Wt and Tg mice. No significant difference was found between Wt and Tg mice. B) Leakage of creatine kinase (CK) before and after ischemia and 5, 10 and 30 min after reperfusion of Wt and Tg mice. No significant difference was found between Wt and Tg mice. C) Functional recovery; LV developed pressure (LVDP) for Wt and (P)RR Tg hearts after 30 min of reperfusion. D) Rate–pressure product (RPP) for Wt and (P)RR Tg hearts after 30 min of reperfusion. Values are means ± SEM; n = 6 per group.

Isolated hearts from Wt and Tg mice were subjected to 45 minutes no-flow global ischemia at 37°C. As shown in figure 4A and 4B, we assessed myocardial damage by lactate dehydrogenase (LDH) and creatine kinase (CK) in the coronary effluent at different time points. Immediately after ischemia (0 min reperfusion) both LDH and CK influx were significantly increased compared to the stabilization period for both Wt and Tg mice; no differences were observed between the genotypes neither after ischemia nor during the reperfusion at 5, 10, and 30 minutes (figure 4A and 4B). The post ischemic recovery of LVDP and post-ischemic recovery of RPP at 30 minutes reperfusion were also similar between the genotypes (figure 4C and 4D). These data suggest that myocardial overexpression of (P)RR does not protect nor exacerbate ischemia/reperfusion injury.

### *In vitro* (P)RR Overexpression in HeLa Cells and Hypoxia Related Gene Response

**Figure 5:**
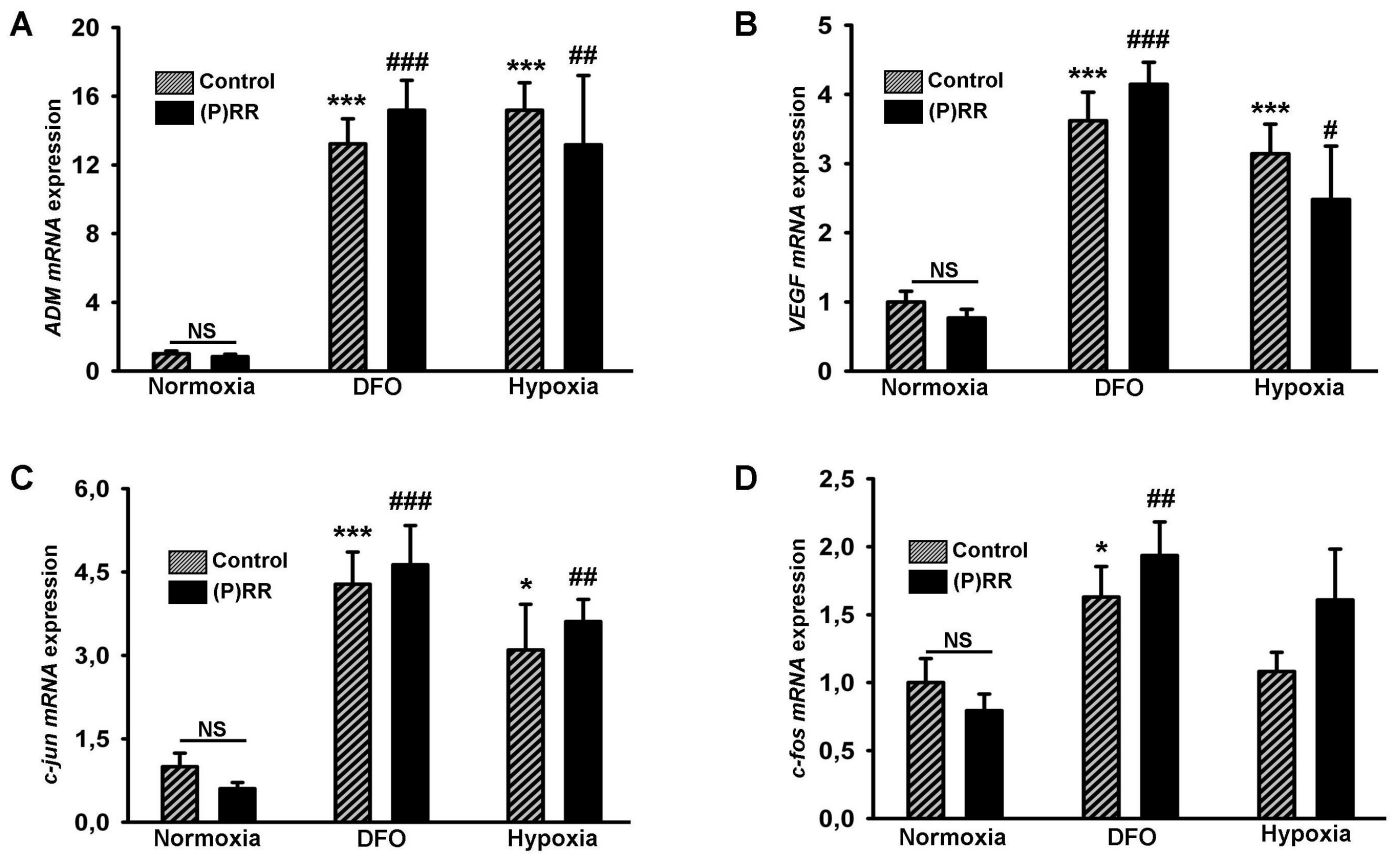
Effect of (P)RR overexpression on stress related gene expression due to hypoxia. Gene expression levels in HeLa cells under normoxia, DFO (deferoxamine) treatment and hypoxia; HeLa cells without (fine bars) and with (black bars) (P)RR overexpression. A) *ADM* B) *VEGF* C) *c-jun* D) *c-fos* (all normalized to *GAPDH*). *P<0.05, ***P<0.001, normoxia vs. DFO or normoxia vs. hypoxia, under control condition. ^#^ *P<0.05,* ^##^ *P<0.01* and ^###^ *P<0.001*, normoxia vs. DFO or normoxia vs. hypoxia, under (P)RR overexpression.

Transient transfection of HeLa cells with (P)RR plasmid leads to a ∼130 fold upregulation of (P)RR mRNA (Figure S1) and ∼5-fold upregulation of (P)RR protein [11]. To examine if (P)RR overexpression has effects on hypoxia we evaluated the expression of genes involved in hypoxia sensing such as *ADM*, *VEGF*, and other immediate early genes (*c-jun* and *c-fos*) in response to hypoxia and the hypoxia mimicking agent deferoxamine (DFO). Real-time PCR analysis revealed a significant increase in expression of *ADM* and *VEGF* mRNA in both DFO and hypoxia conditions (compared to normoxia), but there were no differences in this response between the control HeLa cells and (P)RR overexpressing HeLa cells (figure 5A and 5B). Similarly, *c-jun* and *c-fos* mRNA expression were also increased significantly in DFO and hypoxia treated HeLa cells, but no differences were observed between the control HeLa cells and and HeLa cells overexpressing (P)RR (figure 5C and 5D).

## Discussion

We report the generation of a mouse with cardiorestricted overexpression of the prorenin-renin receptor (P)RR. We demonstrate that this α-MHC-(P)RR mouse has no spontaneous cardiac phenotype, and several established perturbations led to an equal degree of cardiac damage in Tg and Wt mice. We conclude from our data that increased myocardial levels of (P)RR are unlikely to be involved in the development of cardiac remodeling and failure.

Since (P)RR was cloned 11 years ago, there have been many publications on its putative role in cardiovascular and renal disease. Until (P)RR was discovered, it was believed that circulating renin and (pro)renin themselves would be unharmful, as their effects were thought to be conferred via downstream elements of the RAS, specifically angiotensin II and aldosterone [26]. However, binding of renin and prorenin to the (P)RR was shown to exert specific downstream effects in cell culture systems that mimic AngII-induced effects [27], [28], for example DNA synthesis, activation of stress-related kinases (MAPK, ERK), activation of PAI-1 and phosphorylation of heat shock proteins [2], [29], [30]. Furthermore, when (pro)renin or renin bind to (P)RR, renin activity increases, resulting in enhanced signaling of the ‘classical’ RAS route. Thus, high levels of local (P)RR may theoretically have profound impact on local RAS activity.

Most studies addressing the role of (P)RR have focused on renal (patho-)physiology and little knowledge exists on the role of (P)RR in the heart, one of the main target organs of the RAS. Transgenic rats have been generated with ubiquitous (P)RR overexpression and it was reported that no cardiac phenotype was present, although most articles provide very limited data on the cardiac phenotype [8], [9], [15]. Even though beneficial effects of handle region peptide (HRP), a putative (P)RR blocker, have been reported, the efficacy and specificity of HRP has generated contradictory results by other studies [31]–[33] and caused considerable debate in the literature.

On the other hand, deletion of (P)RR, the gene product of the *Atp6ap2* gene, results in a lethal cardiomyopathy phenotype which has been attributed to renin- and prorenin-independent vacuolar H+ATPase and Wnt receptor signaling of this gene [6], [34]. Clearly, the (P)RR is not only a receptor for prorenin and renin, but has other functions as well that are crucial to the organism. Therefore, it is warranted to answer the question if overexpression of (P)RR, as observed in cardiac remodeling [11], is causally involved with this phenotype or rather a consequence of it. Therefore, in the present study, we have investigated the role of myocardial (P)RR overexpression in cardiac remodeling in vivo.

Previous studies in which the human (P)RR was overexpressed in vascular smooth muscle tissue showed that these transgenic rats developed hypertension, which might be associated with the development of nephropathy [15]. Another rat strain in which human (P)RR was overexpressed ubiquitously, showed that these rats developed proteinuria and slowly progressive nephropathy, in the absence of changes in RAS activity suggesting a possible role of (P)RR in kidney disease [9]. Other studies showed that local (P)RR expression is increased in patients with diabetic nephropathy and end-stage kidney disease [35] and animal studies demonstrate up-regulation of renal (P)RR in rat models of hypertension [10], diabetes [36] and renal injury [37]. Increased myocardial expression has been described in the failing hearts of various species and with various etiologies [11] but the precise mechanism of (P)RR contribution in the exclusively heart has remained unclear.

We achieved to generate a transgenic mouse line (one positive founder line out of >22 lines) and this line had a strong overexpression of (P)RR mRNA (∼170 fold), with a more modest increase in (P)RR protein of ∼5 fold, a level comparable to increases in common murine and rat models of cardiac remodeling [11]. This level of overexpression was not associated with spontaneous morphological abnormalities of the heart of Tg mice, while transcriptional analyses did not suggest activation of genes typical for cardiac remodeling, such ANP, BNP and fibrotic genes. ISO infusion, which causes cardiac remodeling via direct effects on the heart in combination with increased renin levels caused equal cardiac hypertrophy and fibrosis in Wt and Tg mice. Also, I/R injury in the isolated heart caused identical injury toWt and Tg mouse hearts. Finally, we studied HeLa cells with and without (P)RR overexpression and could not establish any effect of (P)RR on expression of genes associated with the hypoxic response.

A recent study by Moilanen et al. was the first to specifically address the role of (P)RR in the heart. The authors employed a intramyocardial adenovirus mediated gene delivery of (P)RR which resulted in enhanced matrix remodeling, independent of AngII generation [16]. This was associated with adverse cardiac remodeling. Clearly, our results are in contrast to the observations of this study. The differential outcomes may be explained by the methodology. First, Moilanen et al. used adenoviral mediated transfection, and they showed high levels of overexpression after 3 days and 1 week (up to 7-fold), that decreased over the course of weeks. We achieved stable overexpression of about 5-fold, throughout the entire life span of the mice. Second, their method delivered (P)RR throughout the entire left ventricle. The authors showed predominant myocytic expression, however the method of delivery may have rsulted in delivery to cardiac fibroblasts, not just myocytes, so that the effects on the extracellular matrix may be explained. They do not show if (P)RR was expressed by extracardiac tissue, which could also have played a role. In our study, we overexpressed (P)RR in cardiomyocytes only which easily explains, absence of fibrotic effects. The absence of signaling via (P)RR was paralleled by the observations we made in a cell system.

As discussed, (P)RR may not be essential for cardiac remodeling, but previous data suggest an important role for the embryonic development and organogenesis, especially for the heart [6]. Cardiospecific (P)RR knock out animals are not viable. Batenburg *et. al*. recently showed that direct interaction between prorenin or renin with the (P)RR *in vivo* is very unlikely to occur in non-renin synthesizing organs [38]. Therefore, phenotypes [9], [15] that develop in (P)RR overexpressing transgenic rats are probably largely independent from RAS activity, obscuring the true role of (P)RR in heart. In our studies, we did not assess if renin or prorenin binds directly to the (P)RR which is a limitation of our study. Further, our study only addressed short-term functional effects of (P)RR overexpression; chronic overexpression of the (P)RR, for instance in aged mice, may be associated with structural or functional changes in the heart, but were not assessed in these studies. However, ISO infusion caused an increase of circulating renin, while the response in cardiac remodeling was completely comparable between Wt and Tg mice so that differential renin signaling is improbable. The complete lack of any phenotypic manifestation of the Tg mouse, also in models that have established RAS dependency, supports the notion that cardiac (P)RR has no clear effect on cardiac physiology.

In conclusion, our studies showed that cardio restricted (P)RR overexpression exerts no deleterious effects on cardiac function, on the remodeling process in response to β-adrenergic activation, or on ex vivo ischemia reperfusion injury. Further studies will be essential to fully understand the role of (P)RR in cardiac pathophysiology, but from our data it seems unlikely this role is of major importance.

## Supporting Information

Figure S1 **(P)RR mRNA expression in HeLa S3.** RT-PCR analysis of (P)RR mRNA expression in control HeLa S3 cells (left bar) and (P)RR–overexpressing HeLa S3 cells (right bar) reveals ∼130 fold upregulation in (P)RR levels (normalized to *GAPDH*).
(DOCX)

Table S1 List of primers.
(DOCX)

Table S2 Echocardiographic data and blood pressure measurement.
(DOCX)
