# Supplementary material for: Cardiac Function and Architecture Are Maintained in a Model of Cardiorestricted Overexpression of the Prorenin-Renin Receptor
Source: PLoS One. 2014 Feb 25;9(2):e89929. doi: 10.1371/journal.pone.0089929 (PMC3934958; doi:10.1371/journal.pone.0089929)

**Supplementary Figure 1: (P)RR mRNA expression in HeLa S3.**

RT-PCR analysis of (P)RR mRNA expression in control HeLa S3 cells (left bar) and (P)RR–overexpressing HeLa S3 cells (right bar) reveals ~130 fold upregulation in (P)RR levels (normalized to *GAPDH*).


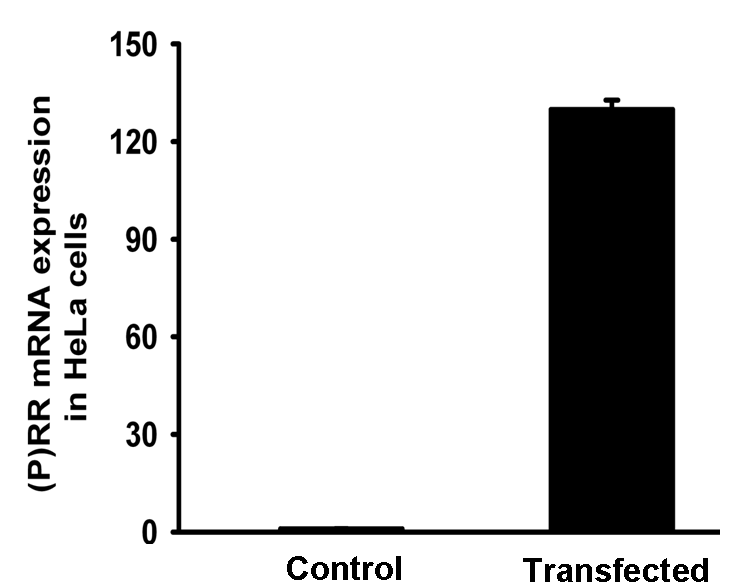

Supplement: Figure S1 — (P)RR mRNA expression in HeLa S3. RT-PCR analysis of (P)RR mRNA expression in control HeLa S3 cells (left bar) and (P)RR–overexpressing HeLa S3 cells (right bar) reveals ∼130 fold upregulation in (P)RR levels (normalized to GAPDH). (DOCX) [file pone.0089929.s001.docx]
