# Supplementary material for: Cardiac Function and Architecture Are Maintained in a Model of Cardiorestricted Overexpression of the Prorenin-Renin Receptor
Source: PLoS One. 2014 Feb 25;9(2):e89929. doi: 10.1371/journal.pone.0089929 (PMC3934958; doi:10.1371/journal.pone.0089929)
Supplement: Table S1 — List of primers. (DOCX) [file pone.0089929.s002.docx]

**Supplementary table 1:** List of primers

| Genes | Forward primers | Reverse primers |
| --- | --- | --- |
| *Mu (P)RR (SalI & HindIII)* | CAAGTCGACCATGGCTGTGCTGGTCGTTCT | CAAAAGCTTTCAATCTATTCGAATCTTCT |
| *Mu (P)RR* | TTCTGAACTGCAAGTGCTGCAT | CTGCCAGCTCCAGTGAATACAAG |
| *Hu (P)RR* | ATTGGCCTATACCAGGAGAG | GGTAGAGCCAGTTTGTTCAC |
| *ANP* | ATGGGCTCCTTCTCCATCAC | TCTACCGGCATCTTCTCCTC |
| *BNP* | GGGCTGTAACGCACTGAAGT | GGAAAGAGACCCAGGCAGA |
| *Gal-3* | TATCCTGCTGCTGGCCCTTATG | GTTTGCGTTGGGTTTCACTG |
| *Col-1* | CTTCACCTACAGCACCCTTGTG | CTTGGTGGTTTTGTATTCGATGAC |
| *Col-3* | GCGATTCAAGGCTGAAG | GGGTGCGATATCTATGATGG |
| *MCIP1* | GCTTGACTGAGAGAGGCGAGTC | CCACACAAGCAATCAGGGAGC |
| *ADM* | TTCCGTCGCCCTGATGTACC | ATCCGCAGTTCCCTCTTCCC |
| *VEGF* | GCCTTGCCTTGCTGCTCTAC | GTCCACCAGGGTCTCGATTG |
| *c-fos* | TCCAAGCGGAGACAGATCAAC | TGGCAATCTCGGTCTGCAA |
| *c-jun* | GATCATCCAGTCCAGCAATG | TATTCTGGCTATGCAGTTCAG |
| *α-MHC* | GTTAACCAGAGTTTGAGTGACA | CCTTCTCTGACTTTCGGAGGTACT |
| *β-MHC* | ATGTGCCGGACCTTGGAAG | CCTCGGGTTAGCTGAGAGATCA |
| *36B4* | AAGCGCGTCCTGGCATTGTC | GCAGCCGCAAATGCAGATGG |
| *GAPDH* | CATCAAGAAGGTGGTGAAGC | ACCACCCTGTTGCTGTAG |
