# Supplementary material for: Cardiac Function and Architecture Are Maintained in a Model of Cardiorestricted Overexpression of the Prorenin-Renin Receptor
Source: PLoS One. 2014 Feb 25;9(2):e89929. doi: 10.1371/journal.pone.0089929 (PMC3934958; doi:10.1371/journal.pone.0089929)
Supplement: Table S2 — Echocardiographic data and blood pressure measurement. (DOCX) [file pone.0089929.s003.docx]

**Supplementary table 2:** Echocardiographic data and blood pressure measurement.

| Parameters | Wt-sham  (N=7) | Tg-sham  (N=7) | Wt-ISO  (N=7) | Tg-ISO  (N=7) |
| --- | --- | --- | --- | --- |
| HR (bpm) | 492.5±25.1 | 434.2±20.2 | 553.7±19.8 | 554.1±13.2^#^ |
| IVSd (mm) | 0.75±0.03 | 0.7±0.02 | 0.98±0.01^**^ | 0.93±0.03^#§^ |
| IVSs (mm) | 1.24±0.03 | 1.3±0.07 | 1.48±0.04^*^ | 1.43±0.05 |
| LVPWd (mm) | 0.67±0.02 | 0.62±0.04 | 0.95±0.04^*^ | 0.92±0.02^#^ |
| LVPWs (mm) | 1.36±0.07 | 1.32±0.03 | 1.54±0.04 | 1.41±0.04 |
| EF (%) | 76.9±1.2 | 76±4.7 | 85±3.6 | 68.8±1.8^§^ |
| FS (%) | 39.8±1.1 | 39.8±3.5 | 49.8±4.3 | 33.4±3.3^§^ |
| LVIDd (mm) | 3.61±0.12 | 3.8±0.15 | 3.55±0.2 | 4.04±0.04 |
| LVIDs(mm) | 2.17±0.09 | 2.3±0.23 | 1.81±0.24 | 2.69±0.06^§^ |
| SBP (mmHg) | 103.5±1.17 | 95.9±3^#^ | 96.8±3.03 | 105.3±2.7 |
| DBP (mmHg) | 73±1.28^¥¥^ | 63.4±1.92^#^ | 69.4±2.92 | 76.8±1.91 |
| MAP (mmHg) | 83.1±1.23^¥^ | 74.2±2.21^#^ | 78.5±2.89 | 86.3±2.14 |

Echocardiographic data before sacrifice. Blood pressure was measured by millar at sacrifice. Data are expressed as means±SEM. HR, Heart Rate; IVSd, InterVentricular Septal width (diastole); IVSs, InterVentricular Septal width (systole); LVPWd, Left Ventricualr Posterior Wall (diastole); LVPWs, Left Ventricualr Posterior Wall (systole); EF, Ejection Fraction; FS, Fractional Shortening; LVIDd, Left Ventricular Internal Dimension (diastole);LVIDs, Left Ventricular Internal Dimension (systole); SBP, systolic blood pressure; DBP, diastolic blood pressure; MAP, mean arterial pressure.

*P<0.05, **P<0.01, sham vs ISO for Wt mice; ^#^P<0.05, sham vs ISO for Tg mice; ^¥^P<0.05, ^¥¥^P<0.01 Wt-sham vs Tg-Sham and ^§^P<0.05, ISO treatment differences between Wt and Tg mice.
